# Processing of Bimetallic Inconel 625-16Mo3 Steel Tube via Supercritical Bend: Study of the Mechanical Properties and Structure

**DOI:** 10.3390/ma16206796

**Published:** 2023-10-21

**Authors:** Igor Barenyi, Martin Slany, Karel Kouril, Jan Zouhar, Stepan Kolomy, Josef Sedlak, Jozef Majerik

**Affiliations:** 1 Faculty of Special Technology, Alexander Dubcek University of Trencin, 911 06 Trenčín, Slovakia; igor.barenyi@tnuni.sk (I.B.); jozef.majerik@tnuni.sk (J.M.); 2 Faculty of Mechanical Engineering, Institute of Manufacturing Technology, Brno University of Technology, 616 69 Brno, Czech Republic; karel.kouril@vutbr.cz (K.K.); zouhar@fme.vutbr.cz (J.Z.); stepan.kolomy@vutbr.cz (S.K.); sedlak@fme.vutbr.cz (J.S.)

**Keywords:** Inconel 625, bimetallic material, welding, mechanical properties, microstructure, supercritical bend

## Abstract

Incineration is currently the standard way of disposing of municipal waste. It uses components protected by high-temperature-resistant layers of materials, such as Inconel alloys. Therefore, the objective of the current paper is to study the mechanical properties and structure of a bimetallic Inconel 625-16Mo3 steel tube. The Inconel 625 layer was 3.5 mm thick and was applied to the surface of the tube with a wall thickness of 7 mm via the cold metal transfer method. The bimetallic tube was bent using a supercritical bend (d ≤ 0.7D). This paper is focused on the investigation of the material changes in the Inconel 625 layer areas influenced by the maximum tensile and compressive stresses after the bend. The change in layer thickness after the bend was evaluated and compared to the non-deformed tube. In addition, the local mechanical properties (nanohardness, Young modulus) across the indicated interfacial areas using quasistatic nanoindentation were investigated. Subsequently, a thorough microstructure observation was carried out in areas with maximum tensile and compressive stresses to determine changes in the morphology and size of dendrites related to the effect of tensile or compressive stresses induced by bending. It was found that the grain featured a stretched secondary dendrite axis in the area of tensile stress, but compressive stress imparted a prolongation of the primary dendrite axis.

## 1. Introduction

In recent years, the incineration of municipal waste has become a favorable alternative to traditional waste landfilling. In addition to waste disposal, incineration has other benefits. The generated heat energy can be used to create electricity or for heating. Despite its advantages, the incineration process also poses a problem that must be solved. The temperature when incinerating ordinary municipal waste is about 800 to 900 °C, and for hazardous waste, temperatures up to 1300 °C are used. The combustion environment is very aggressive, and the components must withstand the high-temperature corrosion caused by Cl, N_2_, O_2_, etc. One of the available solutions is to apply a protective Inconel 625 layer to the component. The component from which the experimental samples were made is part of an incineration chamber for municipal waste. It is a 16Mo3 steel tube through which supercritical cooling water flows, and its outer surface is under the influence of aggressive conditions. The main damage factor is the high-temperature influence (about 500 °C) and subsequent high-temperature corrosion in a chemically aggressive environment. Therefore, the tube is coated with an Inconel 625 layer with resistance to these conditions [1,2]. The layer is made using the cold metal transfer (CMT) method. CMT welding is a modified MIG/MAG welding process based on a short-circuiting transfer process developed by the Austrian welding company Fronius in 2004. This process was developed based on the MIG/MAG welding process but differs in the type of droplet transfer method, which had not been previously encountered [3,4]. The conventional MIG/MAG process uses short-circuiting droplet transfer, while CMT employs a new mechanical droplet-cutting method to transfer molten metal to the weld [2]. Moreover, CTM uses shallow currents that lead to a significant reduction in heat generation. A new type of droplet transfer reduces the Fe content in the clad layer, leading to enhanced coating resistance to high-temperature corrosion [5]. The dissolution of the base metal in the clad layer is almost negligible, resulting in a very narrow fusion zone and a narrower HAZ compared to the MIG/MAG process [5]. The principle, advantages, limitations, and applications of the CMT technique were discussed by Lorenzin and Rutili in detail in their work [4]. During welding, temperature variations in welds and parent metals have important effects on material characteristics and residual stresses, as well as on the dimensional and shape accuracies of welded products [6,7]. The CMT method has been effectively used for the welding and cladding of Inconel, Aluminum, Titanium alloys, steels, and other materials [8,9,10,11,12].

The coated tube is bent by supercritical bending (d ≤ 0.7D; d is the diameter of the bend; D is the diameter of the tube) as part of the manufacturing process of the incinerator component [13,14]. The parameters and mechanical properties of the bend were analyzed, and a specific procedure was developed to cold-bend the tube with the use of sand filling inside to bend it without cracking and excessive deformation of its cross-section [13,14].

**Figure 1:**
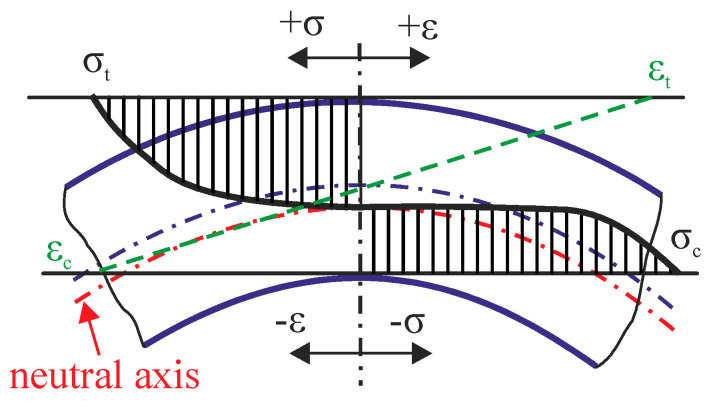
Acting stresses and deformations during the bending process.

Cold bending requires a very high value of the yield strength of the material to cause a shape change in the material, which leads to the plastic deformation of the material. The tensile strength cannot be exceeded to avoid material failure. Internal stresses arise in the material due to cold bending. The acting stresses and deformations during tube cold bending (see Figure 1) were analyzed by several authors using materials including the clad layer of Inconel 625 [15,16,17,18]. During each bending process, tensile stresses (σ_t_) occur in the outer layers of the workpiece relative to the bending radius, and elongation of the material occurs in this area (ε_t_). On the contrary, the inner layers are subjected to compressive stresses (σ_c_), and the material is compressed (ε_c_) in this area. Among them is the so-called non-deformed layer without acting longitudinal stresses, where ε = 0 (neutral axis), and thus, the material retains its original shape and volume. This layer does not copy the theoretical bend radius but is shifted inward in the direction of the bend axis. For this reason, certain elongation occurs during the bending process of the tube, which can be determined by using a mathematical calculation. When bending a hollow tube with a constant wall thickness, the mentioned stresses have an analogous effect on the wall thickness. There is a decrease in the outer radius relative to the theoretical bending radius, and on the contrary, in the inner radius, the thickness of the wall increases.

The authors Guo, X. et al. [19] carried out simulations and real experiments on H62 brass tube bends with different parameters and investigated the influence of these parameters on the bending properties. As part of their experiments, they also investigated the dependence of the bending ratio, R/D_0_ (R—bending radius; D—tube diameter), on the resulting stresses and the ratio of the pipe wall thickness before and after bending. Their experiments proved that with the increase in the bending ratio, the value of internal stresses caused by bending decreased exponentially. Also, the wall thickness ratio between the original tube and the bent tube decreased with the increasing bending ratio R/D_0_ [19]. The authors evaluated the mentioned parameters at the place of maximum compressive stresses in the lower part of the bending arc.

During the cold bending of the tube, plastic deformation of the material occurs, which causes strain hardening. This is a fundamental factor in assessing hardness changes in the areas of the material most affected by bending stresses. The authors Katsuji Tosha et al. [20] directly evaluated the effect of compressive and tensile stresses on the final hardness of bent sheets in a surface layer after the sheet bending of C45 steel. Their results show that hardness values after bending decrease with increasing tensile stresses but increase with increasing compressive stresses. However, work hardening in both areas with tensile stress and compressive stress has a noticeable influence. In the areas where the tensile strain is over 1%, the value of the hardness increment with work hardening is larger than the hardness decrement caused by the tensile stresses themselves [20]. The hardness increases with the compressive stress in the elastic strain stage and increases rapidly after the compressive stress reaches the plastic strain stage due to work hardening. In general, in regions of bends with plastic deformation and subsequent work hardening, whether by tensile or compressive stresses, hardness values increase. The highest hardness values were measured in the area of the highest compressive stresses, where the effect of strain hardening is combined with the action of compressive stresses [20].

Several authors have used nanoindentation to evaluate the nanomechanical behavior and local mechanical properties of layers and coatings [21,22,23,24]. Quasistatic nanoindentation tests involve pushing a diamond-tipped indenter head into a material under either load or displacement control. The indentation response of the subsurface layers of the material can be influenced by a change in its plastic behavior caused by internal stresses [25]. Researchers [25,26] noted the influence of internal stresses on the permanent plastic depth determined after indentation and the contact surface. This influence is smaller when using sharp indentation tips, such as those with Berkovich-type geometry. Also, methods were developed to measure and evaluate internal stresses using nanoindentation [25] based on differences in contact area measurements. These small differences should correspond to internal stresses.

Previous works by the authors’ collective [13,14] were focused on the study of the ability to create critical and supercritical bends in bimetal pipes (i.e., bends smaller than 1D, specifically 0.7D) with Inconel 625 cladding, and this solution has not been published in any other study so far. While these previous studies mainly dealt with the technological parameters of the bending process in relation to the presence of possible defects, this work is focused on the detailed material investigation of the microstructure and nanomechanical properties (nanohardness, reduced modulus) of an Inconel 625 cladding layer in different areas affected by stresses caused by the bending process. The main goal is to obtain a comprehensive overview of the behavior of the layer material during bending with the possibility of further investigating the damage mechanism of the layer by high-temperature and chemically aggressive conditions.

## 2. Materials and Methods

### 2.1. Experimental Materials

**Figure 2:**
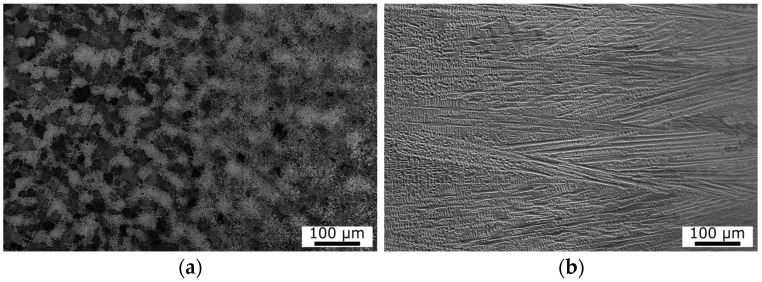
(**a**) SEM microstructure image of 16Mo3 steel (substrate)—the center of the tube wall; (**b**) SEM microstructure image of Inconel 625 clad layer (without bend).

As mentioned above, a bent 16Mo3 steel tube with an Inconel 625 weld overlay was used for experiments. The substrate, 16Mo3 middle-alloyed steel, is heat-resistant steel for use at higher temperatures and pressures. It has good formability, weldability, and corrosion resistance in a water vapor environment up to 530 °C. It is supplied in a state after annealing, where the resulting structure is ferrite and pearlite (Figure 2a). The microstructure was observed in the center area of the tube wall. The basic chemical composition and mechanical properties of 16Mo3 steel are shown in Table 1.

Inconel 625 is a heat-resistant and creep-resistant nickel alloy (NiCr22Mo9Nb). The chemical composition and mechanical properties of Inconel 625 are listed in Table 2. The alloy is substitutionally strengthened by Ni, but Nb and other present elements can cause additional precipitation strengthening. The basic microstructure of Inconel 625 cladding is homogeneous, containing only grains of γ solid solution (Figure 2b) in the form of dendrites. There are also some secondary phases—precipitates of γ’ and γ’’ on the grain boundaries [27]. The Nb precipitates in the γ” phase, while Ti and Al form the γ’ phase. The γ” phase contributes more to the hardening effect than the γ’ phase [28]. The microstructure and mechanical properties of Inconel 625 superalloy are described in more detail by other authors [29,30].

Inconel 625 wire, 1 mm in diameter, was used to overweld the layer on the substrate using the CMT method. The diameter of the basic tube was D = Ø38 mm, while its wall thickness was 7 mm. Additionally, the overwelded outer layer of Inconel 625 had an average thickness of 3.5 mm.

### 2.2. Experimental Sample Preparation

**Figure 3:**
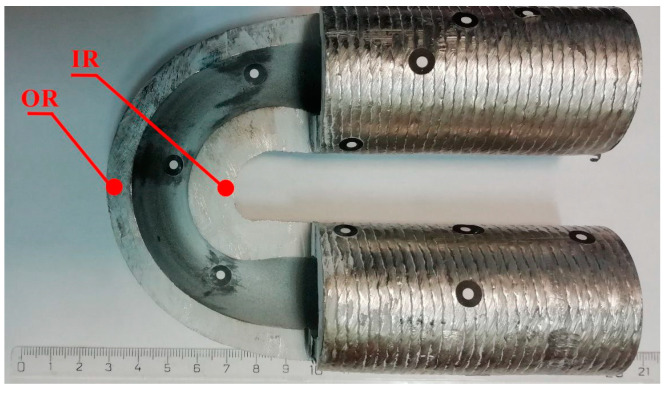
Cross-section of the tube part used for experimental sample preparation.

The tube was bent using a supercritical bend, where d ≤ 0.7D (d is the diameter of the bend; D is the diameter of the tube) at ambient temperature with an applied bending moment of 25.5 kNm. The cross-section of the bent tube part used for the preparation of the experimental sample is shown in Figure 3. The samples were taken from two areas with the greatest internal tensile and compressive stresses after bending (see Figure 1). The place with maximum tensile stresses, marked as OR (outer radius), is located at the top of the bending arc. The place with maximum compressive stresses, marked as IR (inner radius), is located at the bottom of the bending arc.

The samples prepared from the marked places were then processed by using a standard metallographic sample preparation procedure, including pressing into a bakelite mixture, grinding, polishing, and etching. Glyceregia (50% HCl, 33% glycerol, and 17% HNO_3_) was used as an etchant for the Inconel 625 layer microstructure. The final samples were used both for nanoindentation measurement and for light microscopic structural observations. Light microscopic analysis, including some quantitative microscopy parameters (dendrite dimensions), was performed using a confocal laser measurement microscope Olympus Lext OLD5100, Olympus IMS, Webster (Olympus, Trencin, Slovakia) and its analytical software—Stream version 2.4. The structures influenced by tensile and compressive stresses were observed via the ULTRA PLUS scanning electron microscope (Carl Zeiss, Hombrechtikon, Switzerland). EBSD analysis was performed via EBSD NORDLYS (Oxford Instruments, Abingdon, England), and NANO Binning was set to 4 × 4. The measuring EHT voltage was set to 20 kV with a spot size of 120 µm, and the current was 3 nA. The scanning area of the samples was, in all cases, 1 × 0.75 mm.

### 2.3. Quasistatic Nanoindentation

**Figure 4:**
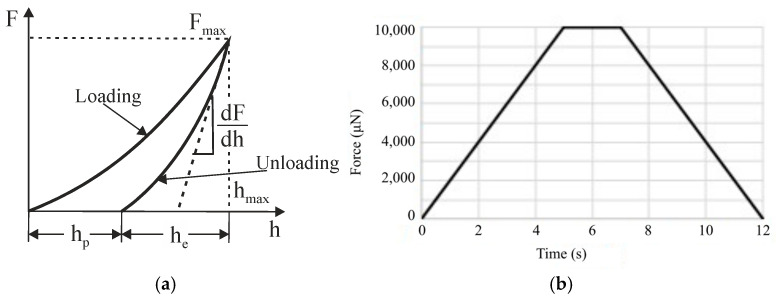
(**a**) Nanoindentation curve of real material and (**b**) loading curve used for the experiment.

Quasistatic nanoindentation is used to measure the courses of nanohardness and the reduced Young modulus across Inconel layers from the surface to a certain depth. The method was first introduced by Oliver and Pharr [31] and is based on pushing a diamond-tipped indenter head into a material, where the displacement (*h*) as a function of the load (*F*) is monitored during both the loading and unloading cycles of the indentation process. The resulting relation *F-h* is called the nanoindentation curve (Figure 4a). The loading part of the curve is used to evaluate nanohardness H, which is defined as the contact pressure under the indenter [32]:

(1) $$H=\frac{F}{A_{c}}$$

where *F* is the load, and *A_c_* is the projected contact area calculated at a depth of indentation h. The unloading part is related to recovering elastic deformation and can be used to calculate the Young modulus of the material.

The initial slope (*S*) of the unloading curve can be related to the elastic modulus of the material using the equation [32]:

(2) $$S=\frac{dF}{dh}=\frac{2E_{r}\sqrt{A_{c}}}{\sqrt{\pi }}$$

where *S* is the initial slope of the unloading curve or contact stiffness, *F* is the applied load, and *E_r_* is the reduced Young modulus.

The conventional Young modulus of the sample (*E_s_*) can be related to the reduced modulus (*E_r_*) using Equation (3), provided that the indenter modulus (*E_i_*) and Poisson’s ratios of the specimen and indenter (*ν_s_* and *ν_i_*, respectively) are known or can be estimated [32]:

(3) $$\frac{1}{E_{r}}=\frac{1-\nu_{s}^{2}}{E_{S}}+\frac{1-\nu_{i}^{2}}{E_{i}}$$

Quasistatic nanoindentation measurements were performed with a Hysitron Triboindenter TI950 (Materials Research Laboratory, Urbana, IL, USA), where the experimental results were analyzed using its software, Hysitron Triboscan version 1.0. A standard trapezoid with maximum force F = 10,000 µN and holding time t = 2 s was chosen as the loading curve for every realized indentation point, as is shown in Figure 4b. A Berkovich indentation tip was used for the experiment, which has sufficient sharpness for the used experimental materials. The number of nanoindentation points was chosen specifically for each Inconel layer area type separately in a way to cover the entire thickness of the layer in the area. The step (distance) between points was constant—150 µm.

## 3. Results

### 3.1. Change in Layer Depth after Bending

**Figure 5:**
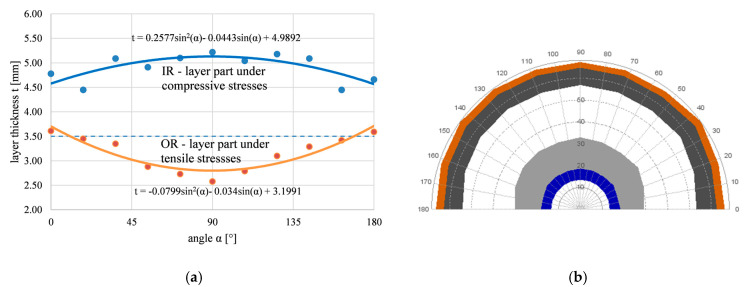
Inconel layer thickness depends on the position angle in the bending arc (**a**), (**b**) the dependence displayed in polar coordinates, including total wall thickness changes.

The change in the thickness of the welded layer due to bending was analyzed as part of the experiments, too. The measurement was carried out with a light microscope and its evaluation software on macrostructure photographs. The achieved results are shown in Figure 5a. Figure 5b shows the results in polar coordinates, including changes in the total wall thickness of the tube. The polar coordinate view displays the complete scheme of the bending arc and allows the visualization of the tube wall deformation, including Inconel layers on both sides of the tube. The regression equations modeled on the basis of experimental data of thicknesses (Figure 5a) clearly indicate the maximum or minimum thickness at the top of the bend, where α = 90°.

As a result of bending, the welded Inconel layer on the outer arc of the bend became thinner due to the tensile stresses present. The maximum thinning is at the top of the outer arc (α = 90°, 73% of the original thickness of the layer). A sample labeled OR was taken from this area. In contrast, the thickening of the Inconel layer occurred due to the presence of compressive stresses in the lower bend arc. The Inconel layer on this side of the arc of the bend has the maximum thickness (α = 90°, 149% of the thickness of the original layer). A sample labeled IR was taken from this area of the bending arc.

The layer thickness changes after bending are related to the types of internal stresses and their distribution within the Inconel 625 layer.

### 3.2. Mechanical Properties across Inconel 625 Layers

**Figure 6:**
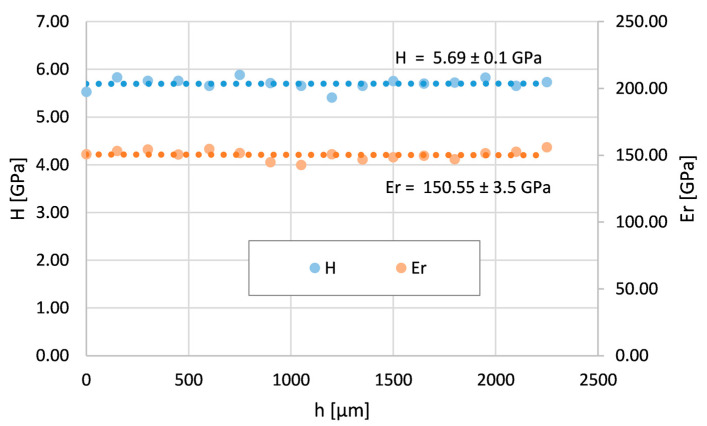
Nanohardness and reduced Young modulus courses across the Inconel 625 layer on the tube without bend (NB).

The courses of the local nanohardness H and reduced Young modulus E_r_ were measured across the Inconel 625 layer of the tube without a bend (NB) and the tube with the bend in areas of maximum tensile stresses (OR) and maximum compressive stresses (IR). It can be assumed that hardness values will be influenced by internal stresses through indentation depth measurements during the nanoindentation process. The measurements do not include the interface between the layer and the substrate or the step change in the local mechanical properties with the values of the substrate. The measurements of the tube without the bend are shown in Figure 6. Both nanohardness and the reduced Young modulus are constant in the entire measured layer cross-section. This was also proven by a regression analysis of the data. The layer is homogeneous without changes in H or E_r_.

The bent tube was analyzed by nanoindentation in two areas (see Figure 3). The first area is at the top of the bending arc (OR), where the Inconel 625 layer thickness is minimum, which means maximum tensile stresses. The second area is at the bottom of the bending arc (IR), where the Inconel layer thickness is maximum due to maximum compressive stresses.

**Figure 7:**
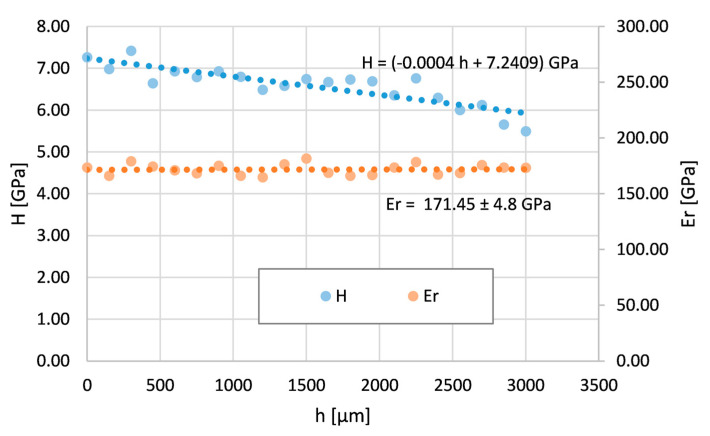
Nanohardness and reduced Young modulus courses across the Inconel 625 layer on the bent tube—the area with maximum tensile stresses (OR).

Figure 7 shows the course of nanohardness and the reduced Young modulus across the area OR. The reduced modulus is also constant in this case. This indicates the absence of any phase transformation or the formation of a new phase across the layer. However, the nanohardness H decreases from the surface. Its values close to the surface are bigger and gradually decrease with depth to the values measured on the tube without the bend.

**Figure 8:**
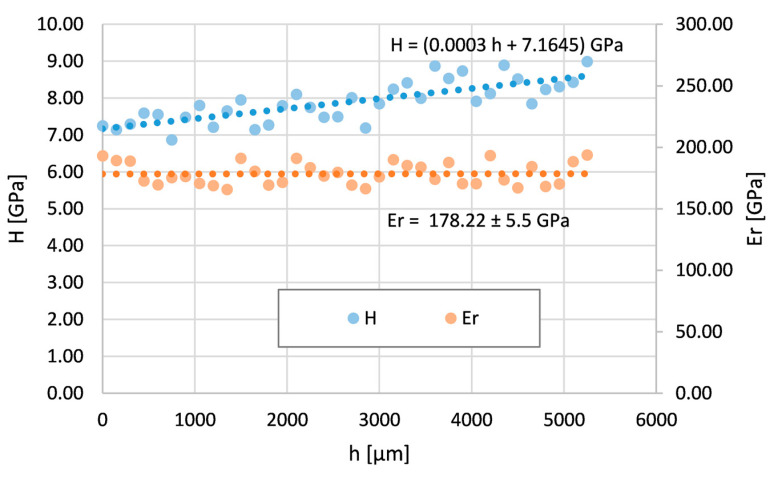
Nanohardness and reduced Young modulus courses across the Inconel 625 layer on the bent tube—the area with maximum compress stresses (IR).

Figure 8 shows the course of nanohardness and the reduced Young modulus across the IR area. The reduced modulus is again constant. The nanohardness H increases from the surface to the deeper areas. Its values gradually increase from the surface and almost double in value compared to the nanohardness of the tube without the bend.

### 3.3. Analysis of Clad Layer Microstructure after Bending

**Figure 9:**
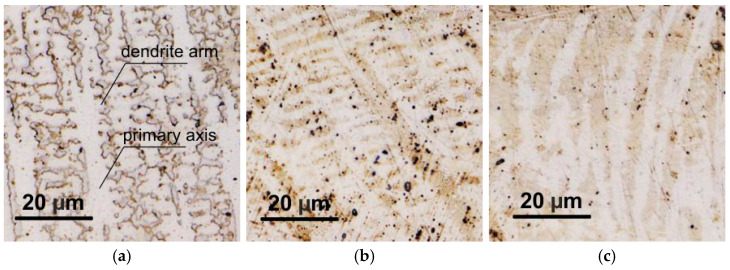
Microstructure of Inconel 625 clad layer and its change after bending. (**a**) Sample without bend (NB), (**b**) area of maximum tensile stresses (OR), and (**c**) area of maximum compressive stresses (IR).

The microstructure of Inconel 625 layers affected by the bending process is documented in Figure 9, which was generated using a confocal microscope (Olympus Lext OLS5100, Olympus IMS, Webster, TX, USA). The structure of the original layer unaffected by bending is shown in Figure 9a to compare changes after bending in the OR (Figure 9b) or IR area (Figure 9c). As described in more detail in Section 2.1, the microstructure of the layer is primarily formed by dendrites of the γ phase. The shape and morphology of dendrites are the most significant indicators of microstructural changes due to bending.

In the area where tensile stresses are acting, the dendrites are stretched in the direction of their secondary axes, called arms (see Figure 9b). The primary dendrite axes are also narrower but remain straight, without deformation or curvature. In the area of compressive stresses (depicted in Figure 9c), the microstructure is noticeably more affected compared to OR. The arms of the dendrites are almost indistinct as a result of deformation by compressed stresses. The dendrite axes are no longer straight but bent with a certain curvature.

The mentioned conclusions are also confirmed by the quantitative microstructure analysis performed using the analytical software Stream of the Olympus Lext OLS5100 (Table 3). The original microstructure photographs were processed in order to highlight dendrite boundaries through their transformation to the two-color scheme, which is most suitable for the quantitative analysis. After processing, the dendrites were more pronounced with sharper borders. The interdendritic space was simplified to a matrix, neglecting the presence of secondary phases. The geometric parameters of the dendrites (width, length) were then evaluated. In the case of the IR region, the length of the arc in which the dendrites were deformed was considered to be the length of the dendrite. Additionally, phase analysis was performed to determine the share of dendrites and the matrix.

The width of the dendrites follows the type of applied stress. It is significantly larger in the tensile area and, conversely, smaller in the pressure area. The lengths of the dendrites do not change significantly in individual investigated cases, even though, in the IR region, they are deformed into the shape of an arc. In terms of the phase share, there was a decrease in the proportion of the interdendritic space (matrix) in both after-bending cases compared to the state without bending. In two after-bending cases, dendrites are differently deformed according to the applied stress. However, the share of the dendritic area is almost the same in both cases.

**Figure 10:**
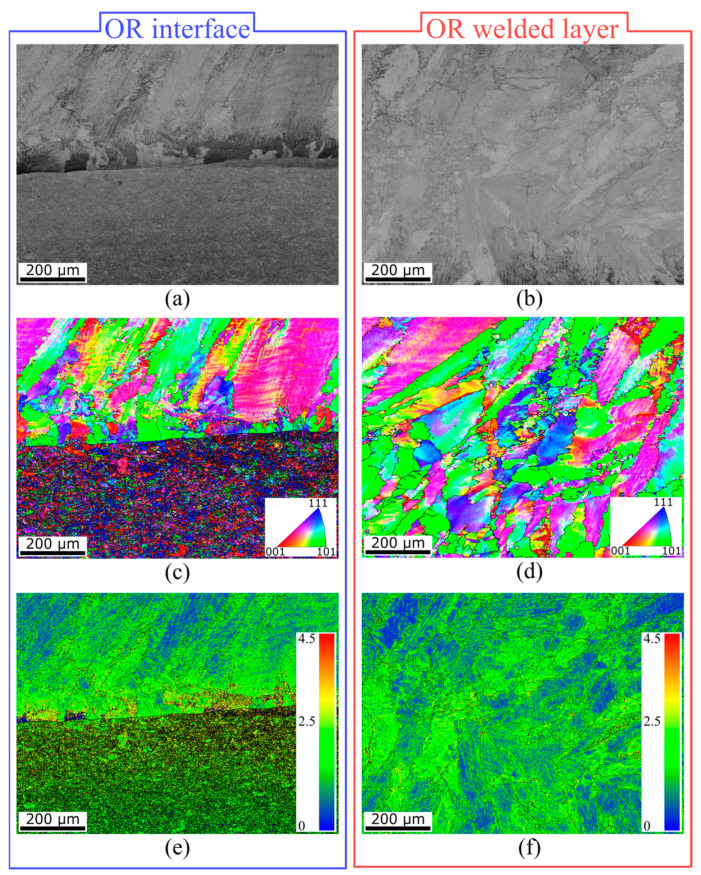
Microstructure of welded Inconel 625 layer and material interface. (**a**) SEM image of the OR interface, (**b**) SEM image of the OR Inconel 625 layer, (**c**) OIM of the OR interface, (**d**) OIM of the OR Inconel 625 layer, (**e**) KAM parameter of the OR interface, and (**f**) KAM parameter of the OR Inconel 625 layer.

SEM analysis was performed in the OR and IR areas for material interface observations. The SEM image of the OR area is depicted in Figure 10a, where the material interface is clearly visible. No visible pores or voids were noted within the structure, which contributed to the appropriate quality of the bimetallic interface. Furthermore, the quality was also confirmed, with no additional cracks in the 625 layer (shown in Figure 10b). Detailed information is provided via orientation image maps (OIMs) captured from the OR interface (see Figure 10c) as well as from the OR welded Inconel 625 layer (see Figure 10d). The orientation of each individual grain is attributed to a color according to the Miller indices depicted in triangles in the figures. The orientations of the grains were evaluated perpendicular to the axis of the welded tube. The grains exposed to the tensile stresses exhibited a stretched secondary axis of the dendrites in comparison to the NB area. In addition, the structure of Inconel 625 featured a randomized grain orientation, while the substrate material showed a preferential orientation to the 111 plane. Figure 10e,f show the Kernel average misorientation (KAM) maps of the OR interface as well as the OR welded Inconel 625 layer. The average grain misorientation angle regarding the adjacent grains can be calculated via the KAM parameter. The average degree of deformation at the OR interface was 0.32° and 0.45° in the case of Inconel 625 and substrate steel, respectively. Furthermore, in the OR welded layer (consisting of Inconel 625), the average KAM parameter was measured at 0.34°. It can be stated that the Inconel 625 material underwent less deformation in comparison to the steel substrate.

**Figure 11:**
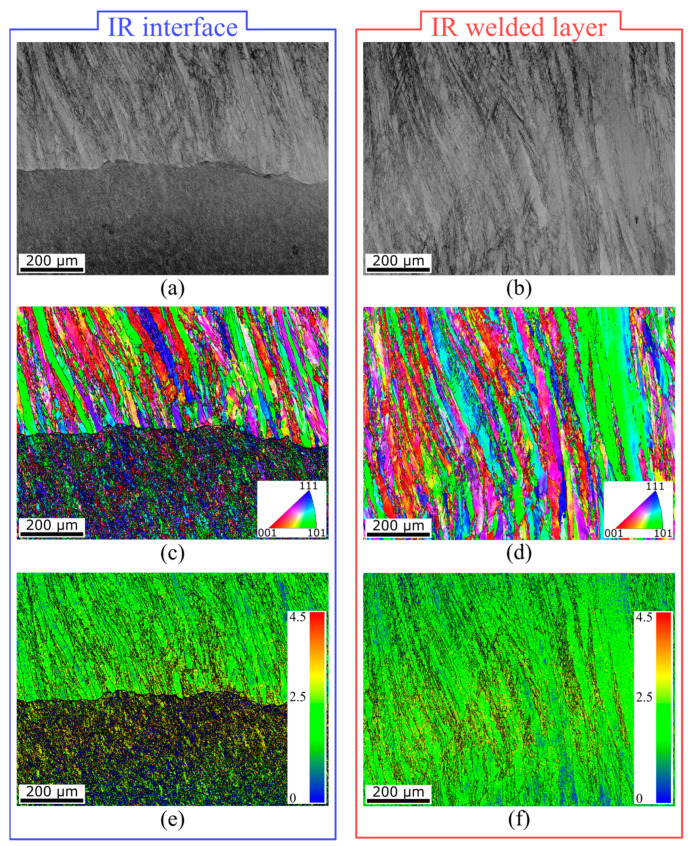
Microstructure of welded Inconel 625 layer and material interface. (**a**) SEM image of the IR interface, (**b**) SEM image of the IR Inconel 625 layer, (**c**) OIM of the IR interface, (**d**) OIM of the IR Inconel 625 layer, (**e**) KAM parameter of the IR interface, and (**f**) KAM parameter of the IR Inconel 625 layer.

The IR interface, as well as the IR welded layer, did not feature any visible cracks, pores, or voids, which was confirmed by thorough SEM analysis, visualized in Figure 11a,b. The absence of these defects means that the designed bending technology has been mastered and the bend is correctly applied. The OIMs of interest were focused on a 1 × 0.75 mm area and were derived from the SEM images. The IR interface depicted in Figure 11c shows a different grain shape than in the OR area. The shape of substrate material grains is more or less similar to that in the OR area, but the primary axis of the grains of Inconel 625 (depicted in the IR welded layer, see Figure 11d) changed to a prolonged shape, and their main axis was longer than in the OR and NB areas. This phenomenon is attributed to the dominant compressive stresses in this area. In regard to grain orientations, the grains also featured no preferential orientation in the OR area. The KAM parameter is depicted in Figure 11e (IR interface) and f (IR welded layer). The average grain misorientation featured the opposite tendency to the OR area. KAM had a higher value (0.42° on average) in the case of Inconel 625, while the steel substrate exhibited a lower value of 0.37°. The opposite trend can be explained by the compressive stresses in the IR area rather than the tensile stresses in the OR area.

## 4. Discussion

As described above, the OR of the bimetallic material is affected by tensile stress and the IR area is affected by compressive stress as a consequence of bending. Furthermore, the ratio of the original change in thickness of the Inconel 625 layer after bending can be interpreted as a relative indicator of the amount of deformation in the areas of the layer. These stress types and the resulting strain are two main factors determining microstructural changes and the subsequent mechanical property distribution within the layer.

**Figure 12:**
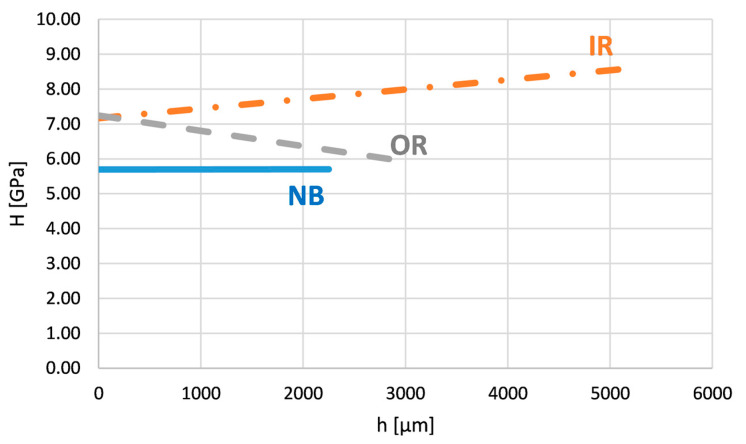
Nanohardness courses across the Inconel 625 layer on the bent tube—the all examined areas i.e., IR, OR, and NB.

Figure 12 shows a summary comparison of the nanohardness curves of all three compared types of layers. It is clear from this figure that the layer without bending (NB) has a constant value of nanohardness throughout the monitored depth. The layer after bending with the largest share of tensile stresses (OR) has a decreasing trend of nanohardness from the surface to deeper subsurface layers. The course of hardness corresponds to the deformation rate of the layer due to bending, while in this case, the largest deformation is on the surface and decreases away from the surface.

In the case of the layer on the inner side of the bending arc (IR), the situation is the opposite. This is the place with the largest share of compression stresses. The hardness in this area (IR) increases slightly from the surface toward the subsurface layers in accordance with the amount of deformation of the layer after bending in this region.

The courses of the reduced (Young’s) modulus of elasticity of all three compared types of layers were constant and did not change depending on the depth below the surface. It assumed that the bending of the layers and the mechanisms associated with it did not initiate any phase transformation changes. The differences in the values of the elastic modulus in the OR and IR areas on one side (170–180 GPa) and NB on the other side (approx. 150 GPa) can be attributed to the influence of some deformation strengthening caused by the bending process. The material changes in the layer hardness are related to detected changes in the shape and morphology of γ dendrites. The results of the presented investigation also correspond to the authors’ previous study of the strength properties in various areas within the whole substrate layer system, including the heat-affected zone [13].

The pipe covered with the protective Inconel 625 layer will be exposed to high-temperature conditions in operation, where the main damaging factor is high-temperature intracrystalline corrosion [33]. The corrosion resistance of the layer also depends on the size and shape of the dendrites, where a finer microstructure means higher corrosion resistance. Therefore, changes in the layers after bending also significantly impact corrosion resistance. The next solution for applying the protective layer is cold dynamic spray deposition [34], which will be our objective in the next study.

## 5. Conclusions

The subject of this research was the weld overlay of heat-resistant Inconel 625 alloy on the 16Mo3 steel substrate. For this research, a semi-finished product was used—a tube with a coating on which a supercritical bend of 180° was created. In the experimental part, the layer without bending was compared with the areas of the bent layer under the influence of maximum tensile or compressive stresses. The changes in the layer depth were first mapped; then, the course of nanohardness and the local modulus of elasticity were evaluated across the layers with a subsequent microstructure analysis in the indicated locations. The investigation can be summarized by the following conclusions based on performed experiments:

- The structure of the coating was homogeneous and formed by a solid solution γ. This was confirmed by the constant course of hardness and reduced modulus of elasticity of the Inconel 625 layer without bending.
- The nanohardness exhibited a decreasing character from the surface in the case of the Inconel 625 layer affected by tensile stresses, while nanohardness featured a slightly increasing character from the surface in the case of the Inconel 625 layer affected by compressive stresses.
- All measured values of nanohardness and the reduced Young modulus for both areas of the bent tube were slightly higher compared to the layer without bending. This increase was caused by deformation strengthening and a related increase in the dislocation number, confirmed by the average KAM parameter.
- The deformation of dendrites and the changes in their size and morphology were related to the applied stress type. Tensile stress resulted in the stretching of the secondary dendrite axes (arms). Compressive stress also caused the deformation of the primary dendrite axes in addition to the narrowing of the arms.

The investigated pipe with the Inconel 625 layer is part of a component used in the incineration of municipal waste. The presented investigation is part of a wider research scope with the aim of increasing the service life of this component, and the results obtained will contribute to the fulfillment of this goal. The studied properties of the layer are also an important factor influencing its corrosion resistance and the layer’s damage mechanism in high-temperature conditions.

## Figures and Tables

**Table 1:** Chemical composition and mechanical properties of 16Mo3 steel.

	wt.%	C	Mn	Si	Mo	Al	P	S	Fe
16Mo3 steel	min.	0.1	0.5	0.15	0.25	--	--	--	Balance
	max.	0.20	0.8	0.37	0.35	0.015	0.04	0.04	
	Tensile strength R_m_ (MPa)			Yield point R_p0,2_ (MPa)			Ductility A (%)		Hardness HB
	440			380			30		150

**Table 2:** Chemical composition and mechanical properties of Inconel 625.

	Wt.%	Cr	Mo	Co	Nb	Ti	Fe	C	Mn	Si	Al	P	S	Ni
Inconel 625 (NiCr22Mo9Nb)	min.	20	8	--	3.15	--	--	--	--	--	--	--	--	Balance
	max.	23	10	1	4.15	0.4	5	0.1	0.5	0.5	0.4	0.015	0.015	
	Tensile strength R_m_ (Mpa)				Yield point R_p0,2_ (Mpa)				Ductility A (%)			Hardness HV		
	965				490				50			200		

**Table 3:** Quantitative microscopy parameters of investigated dendrites.

Parameter	NB	OR	IR
Dendrite width (µm)	9.93 ± 1.11	17.7 ± 0.8	4.75 ± 1.24
Dendrite length (µm)	51.39 ± 1.68	48.94 ± 2.8	52.72 ± 7.45
Area of the dendrites (%)	42.38	54.03	56.75
Matrix (%)	57.62	45.97	43.25

## Data Availability

Data will be made available on request.
